# Beneficial effect of the oxygen free radical scavenger amifostine (WR-2721) on spinal cord ischemia/reperfusion injury in rabbits

**DOI:** 10.1186/1749-8090-4-50

**Published:** 2009-09-17

**Authors:** Fany Chronidou, Efstratios Apostolakis, Ioannis Papapostolou, Konstantinos Grintzalis, Christos D Georgiou, Efstratios N Koletsis, Menelaos Karanikolas, Panagiotis Papathanasopoulos, Dimitrios Dougenis

**Affiliations:** 1 Cardiothoracic Surgery Department, Medical School, University of Patras, Patras, Greece; 2 Biology Department, University of Patras, Patras, Greece; 3 Department of Anaesthesiology and Critical Care Medicine, School of Medicine, University of Patras, Greece; 4 Neurology Department, University of Patras, Patras, Greece

## Abstract

**Background:**

Paraplegia is the most devastating complication of thoracic or thoraco-abdominal aortic surgery. During these operations, an ischemia-reperfusion process is inevitable and the produced radical oxygen species cause severe oxidative stress for the spinal cord. In this study we examined the influence of Amifostine, a triphosphate free oxygen scavenger, on oxidative stress of spinal cord ischemia-reperfusion in rabbits.

**Methods:**

Eighteen male, New Zealand white rabbits were anesthetized and spinal cord ischemia was induced by temporary occlusion of the descending thoracic aorta by a coronary artery balloon catheter, advanced through the femoral artery. The animals were randomly divided in 3 groups. Group I functioned as control. In group II the descending aorta was occluded for 30 minutes and then reperfused for 75 min. In group III, 500 mg Amifostine was infused into the distal aorta during the second half-time of ischemia period. At the end of reperfusion all animals were sacrificed and spinal cord specimens were examined for superoxide radicals by an ultra sensitive fluorescent assay.

**Results:**

Superoxide radical levels ranged, in group I between 1.52 and 1.76 (1.64 ± 0.10), in group II between 1.96 and 2.50 (2.10 ± 0.23), and in group III (amifostine) between 1.21 and 1.60 (1.40 ± 0.19) (p = 0.00), showing a decrease of 43% in the Group of Amifostine. A lipid peroxidation marker measurement ranged, in group I between 0.278 and 0.305 (0.296 ± 0.013), in group II between 0.427 and 0.497 (0.463 ± 0.025), and in group III (amifostine) between 0.343 and 0.357 (0.350 ± 0.007) (p < 0.00), showing a decrease of 38% after Amifostine administration.

**Conclusion:**

By direct and indirect methods of measuring the oxidative stress of spinal cord after ischemia/reperfusion, it is suggested that intra-aortic Amifostine infusion during spinal cord ischemia phase, significantly attenuated the spinal cord oxidative injury in rabbits.

## Background

Paraplegia remains the most devastating complication following descending thoracic or thoraco-abdominal aortic surgery, with incidence rate from 4% to 33% [1]. It is known that spinal cord ischemia from hypoperfusion during temporary aorta cross clamping, as well as the sacrifice of some intercostals branches contributing to the form of Adamkiewicz's artery, are the cause of this complication. The clinical evidence that some patients recover with no neurological dysfunction only to develop delayed-onset of paraplegia 1 to 5 day later, suggests that some neurons remain viable after an ischemic attack but may be at risk during reperfusion [2]. Recently it was demonstrated that the mechanism of spinal cord injury after ischemia-reperfusion consists of progressive loss of motor neurons accompanied with a steady decline of motor function [3]. The complexity of this mechanism is focused to the alteration of the ratio between thromboxane and prostacycline production, lipid peroxidation and reactive oxygen species (ROS) production [4,5]. The ROS which produced as a result of glutamate receptor-activated and subsequently mediated pathways, initiates chain reactions and damage cellular macromolecule, including proteins, DNA and lipids, ultimately leading to cell death [6].

Although several endogenous antioxidant enzymes such as superoxide dismutase, glutathione peroxidase, and catalase can detoxify reactive oxygen species (ROS), the overproduction of the latter during the reperfusion of the ischemic segment of spinal cord, can cause oxidative stress followed by cell death [7]. Mechanical and pharmacological methods have been studied but none has been proven effective enough [8]. In studies, many molecular processes have been investigated towards their intervention in spinal cord injury-ischemia, implicating various cellular mechanisms [9]. The reduction of ROS production has always been at the top of this list.

Our hypothesis was that a ROS scavenger with specific characteristics, such as S-2-3 aminopropylaminoethyl phosphorothioic acid, might be infused during experimentally produced temporary descending aorta ischemia and might prevent the spinal cord ischemic cells from the harmful effect of ROS production during the reperfusion phase.

We used Amifostine (S-2-3 aminopropylaminoethyl phosphorothioic acid, known as WR-2721), which has been well documented to offer protection on normal cells during radiotherapy and chemotherapy, particularly in combination with cisplatin administration [10]. To the best of our knowledge, there have been no other studies investigating the direct or indirect protective effects of Amifostine in spinal cord cells during ischemia-reperfusion injury.

## Methods

Eighteen New Zealand white healthy male rabbits weighing 2.1 to 2.8 kg (mean 2.34 ± 0.17 Kg) were used in this study. Animals were housed under Standard Conditions and Guidelines for the Accommodation and Care of Animal used for experimental and other scientific purposes (1999/575/EU) in the Animal Research Laboratory at Patras University.

### Experimental Design/Groups

The animals were divided into three groups. Group I the control group (*n *= 6): The animals underwent the surgical procedure but the aorta was not occluded. Group II (*n *= 6): Aorta was occluded for 30 min, followed by reperfusion for 75 min. Group III (*n *= 6): Amifostine was infused during the second half-time of aorta occlusion. Animals with blood loss (>15 ml), arrythmia, or/and hemodynamic instability (expressed with a decrease of BP > 15 mmHg for more than 1 min), were excluded from the experiment.

### Antioxidant agent

Amifostine (ETHYOL^®^, Schering-Plough, Swiss) was the anti-oxidant used factor. **ETHYOL **is the trihydrate form of Amifostine known chemically as 2- [(3-aminopropyl)amino]-ethanethiol dihydrogen phosphate (WR2721). It is supplied as a sterile lyophilized powder (10 ml vial contains 500 mg of Amifostine on the anhydrous basis) requiring reconstitution with normal saline 0.9% for intravenous infusion.

### Oxidative stress detection reagent

As a detector of oxidative stress Hydroethidine (HYDRIDINE^®^, Glaxo, Bristol, England) was used. This is a reduced form of ethidium bromide [11,12].

### Surgical procedure

The animals were fasted for 12 hours. Sedation was induced by intramuscular Ketamine (KETAMINE HYDROCHLORIDE^®^, Parke-Davis DIV of Warner-Lambert, USA), (50 mg/kg), and Xylazine (XYLAZINE^®^, Bayer HealthCare, Germany), (10 mg/kg) prior to the procedure [13]. Animals' femoral site, back, tail and ears were prepared before placed in supine position and allowed to breathe spontaneously with O_2 _via face mask (FiO_2 _35%). A 22-gauge venous catheter was placed in the marginal ear vein and CEFAZOLINE SODIUM (VIFAZOLINE^®^, Viannex, Greece), (10 mg/kg), was administered as a single dose [14]. A 22-gauge catheter was placed in the central ear artery. The experiment was recorded in 8 phases. Heart Rate, Arterial Blood Pressure and O_2 _Saturation (Siemens, SC 9000 XL) from the tail artery were monitored continuously and recorded before starting the surgical procedure (phase 1), after the insertion of the femoral arterial catheters (phase 2), after the insertion of the Peripheral Dilatation Catheter (phase 3), 15 min after the administration of the reagent (phase 7), and just before the end of the experiment (phase 8). In addition, in groups (II) and (III) measurements were recorded after aorta occlusion (phase 4), prior to release the occlusion (phase 5) and at the onset of reperfusion (phase 6). Sedation was maintained by intravenous administration of Propofol 1% (PROPOFOL^®^, Astra Zeneca, Chershire, UK), (0.6 mg/kg), and Fentanyl (FENTANYL^®^, Sanofi, Sweden), (0.001-0.002 mg/kg), periodically [15]. Ringer's Lactate (RINGER'S LACTATE^®^, Mayrhofer Pharmakeutica Company) was infused at a rate of 4-10 ml/kg/h, maintaining mean blood pressure between 85 to 100 mmHg [16]. Placing a heating pad under the animal and exposing it to a heat lamp maintained animal body temperature.

Arterial blood samples for partial pressure of O_2 _(PaO_2_), partial pressure of CO_2 _(PaCO_2_), pH, full blood count (FBC) and glucose measurements were obtained in all groups prior to the surgical procedure (phase 1), 15 min after the administration of the reagent (phase7), and just before the end of the experiment (phase 8). Serum Ca^2+ ^level measured in all groups at the onset (phase 1) and at the end of the experiment (phase 8).

After the rabbit had been stabilized and heparinized with (150 U/kg) Heparin Sulfate (10 U/ml) (HEPARIN SULFATE^®^, Leo Pharmaceutical, Denmark), its femoral arteries were isolated and cannulated bilaterally with a 22-gauge catheter. The right one was used for the purpose of monitoring the peripheral blood pressure during the experiment. At the left side, a 5.5FR Peripheral Dilatation Catheter with microglide Coating (AGIL/TRAC .035 GUITANT CORPORATION, Santa Clara, USA) was introduced over a guide-wire, using Seldinger technique. The catheter was advanced to the descending aorta up to the level of left subclavian artery. The level had been estimated in a previous experiment with an open procedure and percutaneous angiography [17]. After that, solution of 0.5 ml of Sodium Heparin (10 U/ml) in 10 ml normal saline 0.9% was used for the protection of the catheter.

Peripheral Dilatation Catheter balloon was inflated by the insertion 0.5 ml water for Injection (8 Atm) and aorta occlusion was established for 30 min. Aorta occlusion was verified by the decrease of blood pressure via the arterial catheter in the opposite femoral artery and also by the increase of blood pressure via the ear arterial catheter.

Amifostine was infused via the Peripheral Dilatation Catheter line intra-aortically and proximally to the occluded segment, 15 min prior to the release of aorta occlusion by deflation of the balloon. When 30 min of aorta occlusion was completed, the balloon was deflated and aortic perfusion was restored. For oxidative stress detection, HE reagent was slowly administered (for 4 min) intra-aortically via the Peripheral Dilatation Catheter line, by the onset of reperfusion. After 75 min of reperfusion the animals were sacrificed with lethal doses of Propofol and Fentanyl [15]. Rapid (<2 min) laminectomy was performed by using rib shears (24-101-22 MARTIN, Tuttlingen, Germany), and lumbar spinal cord was harvested, 2 cm distally to 12^th ^rib.

### Animal treatment

Amifostine (ETHYOL), 500 mg (initially dissolved in 10 ml normal saline 0.9%), was administered to the rabbits as a 15-minute intravenous infusion starting 15 minutes after the aorta occlusion (total duration 30 min) through the tip of the peripheral dilatation catheter proximally into the aorta.

Hydroethidine, 4.7 mg/Kg was administered intra-aortically in the descending thoracic aorta (just after the subclavian artery origin), (in 1 ml solution) to the rabbits, via the Peripheral Dilatation Catheter immediately after deflation of the balloon.

### Tissue treatment

Rabbit spinal cord was homogenized with a 2-ml glass-glass Potter-Elvehjem homogenizer in 1:1 tissue wet weight: volume ice-cold phosphate buffer (50 mM, pH 7.8, containing 10 mM sodium cyanide).

### Superoxide radical assay

The method is based on the reaction between superoxide radical and Hydroethidine that results in the formation of the specific product 2-OH-ethidium, the formation rate of which is measured and converted to superoxide radical production rate [18]. 2-OH-Ethidium is estimated after being extracted from the tissue in alkaline acetone, isolated via cation and hydrophobic microcolumn chromatographies and quantified by the use of its fluorescence properties before and after consumption of 2-OH-ethidium by a horsradish peroxidase (HRP)/H_2_O_2 _system (in the presence of DNA). Fluorescence measurements were performed in a quartz microcuvette (internal dimensions 4 × 4 × 45 mm) with its appropriate holder and a Shimadzu RF-1501 spectrofluorometer set at 10 nm excitation/emission slit width and high sensitivity. Superoxide radical concentration is expressed in pmole mg^-1 ^protein (in 75 min).

### Lipid peroxidation TBARS assay

Spinal cord homogenate was assayed by a modified thiobarbituric acid (TBA)-based method [19]. Specifically, up to 0.15 ml sample was mixed with 0.15 ml TBA reagent [0.5% w/v TBA in 20% w/v trichloroacetic acid (TCA) and 0.33 N HCl]. To the resulting mixture was added 2 μl 2% (w/v) of the lipid antioxidant butyl-hydroxyl anisole (BHA, made in absolute ethanol) to prevent artificial lipid peroxidation production during the assay. The mixture was incubated at 100°C for 20 min and brought to room temperature. To that 0.3 ml isobutanol was added, mixed by vigorous vortexing, centrifuged at 15000 *g *for 3 min, and the fluorescence of the upper butanol layer was measured at excitation 535 and emission 550 nm against butanol-treated sample and reagent blanks (0.15 ml sample plus 0.15 ml 20% TCA containing 0.33 N HCl and 0.02% w/v BHA, and 0.15 ml homogenate-buffer plus 0.15 ml TBA reagent containing 0.02% w/v BHA, respectively). Emission fluorescence was converted to malondialdehyde (MDA) equivalents from a standard curve using malonaldehyde bis(dimethyl acetal) (0-2 nM). Measurements were done in a Shimadzu RF-1501 spectrofluorophotometer set at low sensitivity and excitation/emission bandwidth 10 nm. TBARS were expressed in fmol MDA equivalents mg^-1 ^total protein.

### Protein concentration assay

Protein in ~200× diluted sample homogenates was determined by a modification of a CBB-based method [20]. Specifically, 0.063 ml of the homogenate was mixed with 0.02 ml 0.5% (v/v) Triton X-100 and 0.017 ml 6 N HCl. The mixture was incubated at 100°C for 10 min, brought to room temperature and mixed with 0.9 ml 0.033% (w/v) CBB-G250 stock reagent (made in 0.5 N HCl, stirred for 30 min and filtrated through Whatman #1 filter paper by water pump aspiration, and stored in dark) and incubated for 5 min at room temperature. The absorbance of the mixture at 620 nm was converted to protein mg from a 0-0.05 mg BSA standard curve (against appropriate sample and reagent blanks). A Shimadzu UV-VIS 1201 spectrophotometer was used.

### Statistical Analysis

Data represent mean ± one standard deviation. Statistical analysis was carried out using SPSS for Windows software program version 13.0. A single factor analysis of variance (ANOVA) was performed to check for differences between the three animal subgroups. The comparisons of the differences in vital signs and blood investigations within the same group were performed by single factor analysis of variance (ANOVA) with post hoc comparisons (Tukey-Scheffe-Student Newman Keuls Tests) and among the different groups by the One Way Multivariate ANOVA test. Wilcoxon paired sample test was also used to compare two paired data in the same group. Unpaired Student's *t*-test was performed for the non-parametrically analysis of neurological function score. Differences were considered significant at a P value of < 0.05.

## Results

All rabbits survived until time of sacrifice without significant hemodynamic derangements or other complications. Therefore, additional drug support treatment was not considered necessary during the experiment.

### Clinical outcome

Hind limb paralysis was noticed in all animals of Group II. The administration of Amifostine (Group III) improved neurological status because all animals were able to use their hind limbs. Their ability to hop couldn't be assessed due to short period of anesthetic recovery.

### Vital signs

No statistical significant differences in heart rate and O_2 _saturation were noted during the procedure in each group and among the groups (*p *> 0.05). Between the phases of the experiment, as well as during the slow infusion of Amifostine and reagent Hydroethidine infusion, blood pressure records did not show any statistical difference among three groups. However, there was a significant statistical difference between the phases in the Group II and Group III with (*p *= 0.000), which was attributed to the aortic clamping.

### Blood gases

From blood gases measurements there was no difference in pH among the groups but significant statistical difference was recorded in the Group II among the phases (p = 0.01). In this group there was a decrease in blood pH just after the aorta release (7.34 ± 0.048) as compared to (7.44 ± 0.061) and (7.40 ± 0.042) at the onset (phase 1) and at the end of the experiment (phase 8), respectively.

No statistical difference was obtained in pO_2 _and pCO_2 _in the groups and between the groups with an exception in Group III in which, a decrease of pCO_2 _(30.91 ± 7.9) was observed at the end of the experiment (phase 8) as compared to the other phases (phase1 and phase7) of the experiment (43.56 ± 5.2 and 45.08 ± 9.20), respectively with *p *= 0.01.

**Figure 1:**
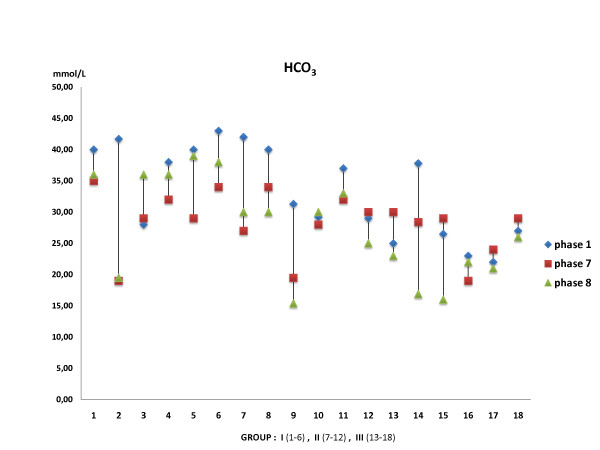
**HCO^3 ^levels**. HCO^3 ^levels graphics shows a decrease in Group III.

Of note, there was a statistically significant difference (*p *= 0.005) between the groups concerning the HCO_3 _^- ^levels, whilst this was not observed within the same group (Figure 1).

### Blood tests

**Figure 2:**
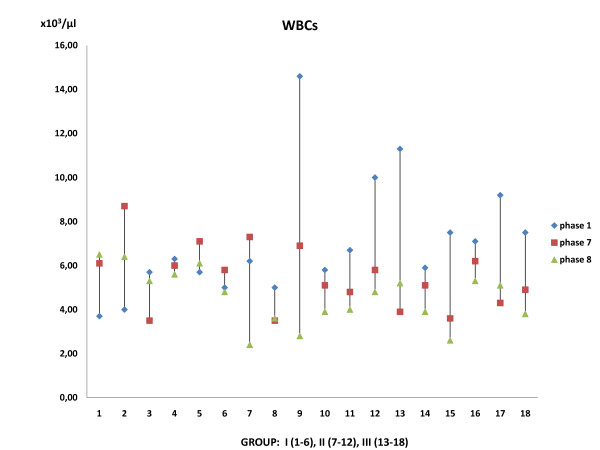
**White blood cells count samples**. Note the statistically significant decrease (*p *= 0.01) of WBCs in Groups (III) and (II) compared with Group (I).

**Figure 3:**
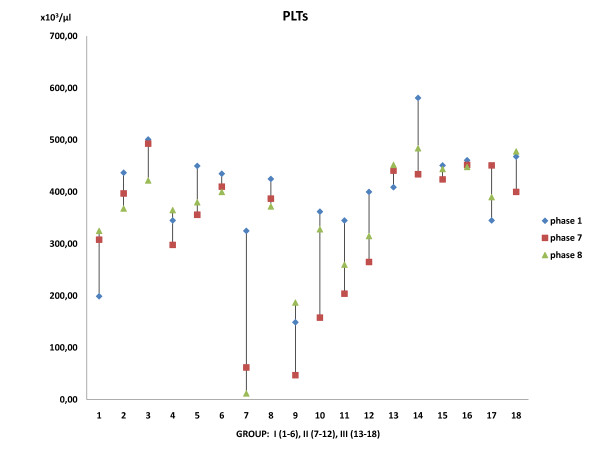
**Platelets count**. A decrease in PLTs is noticed in Group (II) compared with Groups (I) and (III).

Blood results tests obtained at the onset of the procedure, after the aorta release and the administration of the agent and at the end of the experiment revealed the following: 1) No statistically significant difference was observed in Ht between the groups (*p *= 0.058) although there was a difference among the same group, probably due to some blood loss during the operating procedure. 2) Statistically significant decrease in WBC of Groups III and II was observed as compared to Group I (*p *= 0.01) (Figure 2). 3) Statistically significant decrease in the PLTs of Group II was observed as compared with Group I and Group III (*p *= 0.01), although there was no statistical difference within the same group [(*p(I) *= 0.902, *p(II) *= 0.136, *p(III) *= 0.788)] (Figure 3). No statistical difference was noted in serum glucose value throughout the experiment.

**Figure 4:**
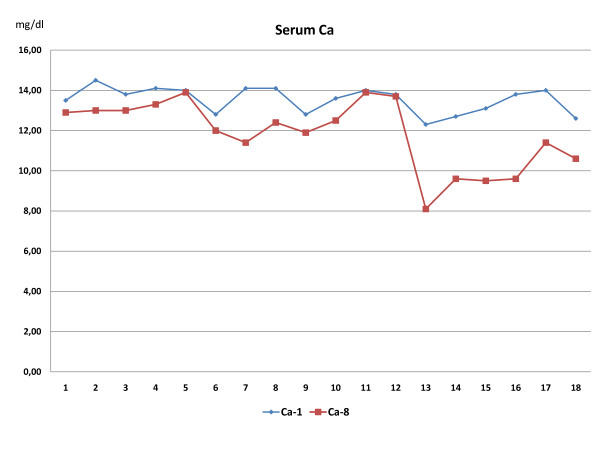
**Serum Calcium levels**. Serum Calcium was measured at the onset (Ca-1) and at the end of the experiment (Ca-8). It's noticed a decrease in Ca levels of 5.5% and 8% in (I) and (II) groups and 25% in (III) (animals 13 to 18).

Serum calcium levels were significantly (*p *= 0.001) reduced at the end of the experiment in all groups (phase 8), with a significant decrease of 25% in Group III of Amifostine (Figure 4).

### Superoxide radical assay

**Figure 5:**
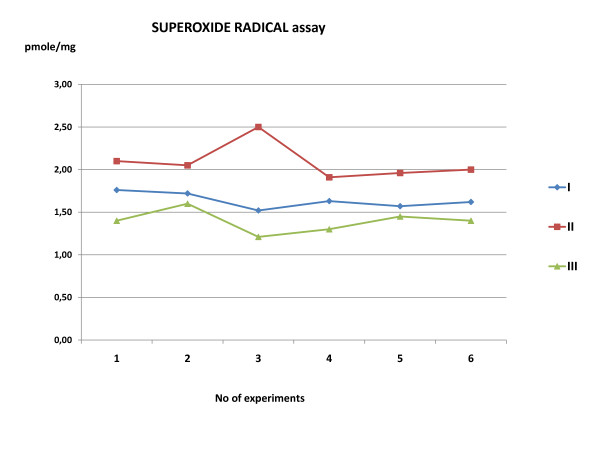
**Superoxide radical assay**. The superoxide radical assay revealed a statistical significant increase (p = 0.000) of 27.43% in superoxide free radical formation in the spinal cord of the ischemic rabbits (Group II) compare to controls (Group I). The values of superoxide radical assay in amifostine group were preserved.

The superoxide radical assay revealed an increase of 27.43% in superoxide free radical formation in the spinal cord of the ischemic rabbits, which was decreased by 42.68% [as much as 15.25% below the Group(I)] by Amifostine administration (Table 1), (Figure 5). Statistically significant difference was found among the groups (*p *= 0.000).

**Table 1:** Superoxide radical assay

**N**	**Control**	**Aorta occlusion**	**Amifostine**
**1**	1.76	2.1	1.4
**2**	1.72	2.05	1.6
**3**	1.52	2.5	1.21
**4**	1.63	1.91	1.3
**5**	1.57	1.96	1.45
**6**	1.62	2	1.4
**Mean ± SD**	**1.64 ± 0.09**	**2.09 ± 0.21**	**1.39 ± 0.13**
**Expressed as %**	**100**	**127.43**	**84.75**

Superoxide radical assay. Superoxide radical (in pmole mg^-1 ^protein for 75 min). ^† ^When a mean value appears it is followed by a standard deviation. ^††^Taking as 100% the mean value of the control group.

### TBARS assay

**Figure 6:**
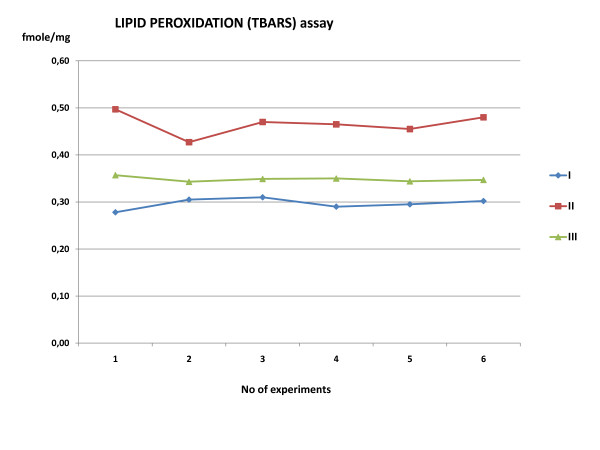
**Lipid peroxidation assay**. TBARS assay demonstrate a statistical significant increase in peroxidation production of 55.3% in Group II compare to controls (Group I). The amifostine administration (Group III) decreased the lipid peroxidation by 35.3%. TBARS: thiobarbituric acid reactive species.

Lipid peroxidation marker TBARS assay results showed an increase in peroxidation production of 55.3% in Group II, which was decreased by 35.3% after Amifostine administration in Group III (Table 2, Figure 6). Statistical analysis showed a significant difference (*p *= 0.000).

**Table 2:** Thibarbituric acid reactive species (TBARS) assay

**N**	**Control**	**Aorta occlusion**	**Amifostine**
**1**	0.28	0.497	0.357
**2**	0.31	0.427	0.343
**3**	0.31	0.470	0.349
**4**	0.29	0.465	0.35
**5**	0.30	0.455	0.344
**6**	0.30	0.480	0.347
**Mean ± SD**	**0.30 ± 0.01**	**0.466 ± 0.024**	**0.360 ± 0.005**
**Expressed as %**	**100**	**155.3**	**120**

Thibarbituric acid reactive species (TBARS) assay (in fmole mg^-1 ^protein). ^† ^When a mean value appears it is followed by a standard deviation. ^††^Taking as 100% the mean value of control group.

## Discussion

For descending thoracic or thoraco-abdominal aorta procedures, during which reduced local tissue perfusion and oxygenation compromise spinal cord function, paraplegia has been considered as the most devastating complication [1,2,21].

The most immediate event at the neuronic cellular level during ischemia, is the depolarization and the consequent opening of voltage-depended ion channels (i.e., Na^+^, K^+^, Ca^+^) [22]. This leads to massive release of a variety of neuro-transmitters including glutamate receptor-operated ion channels. The most important consequence of these rapidly evolving ionic disturbances is the accumulation of intracellular Ca^+^, which initiates several damaging effects/actions. These include [22-24]: a) mitochondrial dysfunction, leading to a failure of aerobic energy metabolism and lactate accumulation, b) activation of mitochondrial and cytoplasmic nitric oxide synthase (NOS) and production of nitric oxide [25], c) activation of phospholipase A_2_, which liberates arachidonic acid (AA), which is then converted by cyclooxygenases (COX 1,2) to a number of deleterious prostanoids and by lipoxygenases (LTs) some of which are chemo-attractants for polymorphonuclear leukocyte and macrophage influx, and) activation of the calcium-activated cysteine protease calpain which is mediating axonal damage in SCI.

One of the consequences of mitochondrial dysfunction, COX and lipoxygenase activity and NOS activation is the formation of reactive oxygen species (ROS), including peroxynitrite anion (ONOO^-^), a product of superoxide radical reaction with nitric oxide [26].

ROS are capable of independent existence. The O_2 _toxicity is due to excess formation of the superoxide radical (O_2 _^-^), a product of the single electron reduction of molecular oxygen [26]. Having too many ROS in relation to the available antioxidants is considered as a state of high oxidative stress, which can cause biomolecular damage. Severe oxidative damage, especially to DNA, may trigger activation of the cysteine protease caspase-3 and consequently death by apoptosis. The onset of apoptosis in oligodendroglia, distant to the site of injury, appears to be unique in acute spinal cord ischemia and contributes to axonal demyelination and dysfunction with long-term neurological deficits.

On the other hand, peroxynitrite anion (ONOO^-^) is capable of causing widespread damage to lipids, proteins and nucleic acids [26]. From these, cell membrane lipid peroxidation has been conclusively demonstrated to be a key mechanism triggering cellular damage. This includes: decreased membrane fluidity which makes it easier for phospholipids to exchange between the two halves of the bilayer, increased membrane leaking to substances that do not normally cross it other than through specific channels (e.g. K^+ ^and Ca^2+^), and damaged membrane proteins and inactivated receptors, enzymes, and ion channels[24,27]. Continued oxidation of fatty acid side chains and their fragmentation to produce aldehydes will eventually lead to loss of membrane integrity, e.g. rupture of lysosomal or central vacuolar membranes. [27,28]

The importance of a treatment strategy is to identify and administer an agent, which can act effectively as a target in the biochemical cascade of apoptosis. This must be a competitive caspase inhibitor with increased cell permeability and sufficient active intra-cellular metabolite level. We showed experimentally by this study that, the organic triophosphate agent Amifostine or WR-2721 appears to be very effective in the reduction of ROS levels produced in spinal cord cells during ischemia-reperfusion injury.

This drug and its trihydrate form is a pro-drug that is dephosphorylated in tissues to a pharmacologically active free thiol. Clinical pharmacokinetic studies showed that it is rapidly cleared from the plasma with a distribution half life of <1 min and an estimated elimination half-life of approximately 8 min. This means that only 10% of ETHYOL remains in the plasma for 6 min after drug administration. In fact, within 15 min after administration it is hydrolyzed by either membrane-bound acid phosphatase or alkaline phosphatase to produce the corresponding free sulfhydryl metabolite WR-1065 [29]. In contrast to the brief plasma half-life, Amifostine and its metabolites are present at maximal levels in tissues between 5 to 15 min following the injection and they also remain intracellularly for long time.

A major advantageous property of Amifostine and its corresponding free thiol WR-1065 is the ability to scavenge free radicals, and to affect cellular DNA repair enzymes and the cell cycle progression. Therefore, this drug is considered as a radioprotective and chemoprotective agent, with antimutagenic, anticlastogenic and antitransforming properties [30]. In addition, it has also been shown that Amifostine can normalize hypercalcemia through its PTH-independent inhibitory effect on TRCa. The key role for this effect is attributed probably to its phosphate group that bounds to or is liberated from the molecule within the extra- and/or intracellular space [31,32].

**Figure 7:**
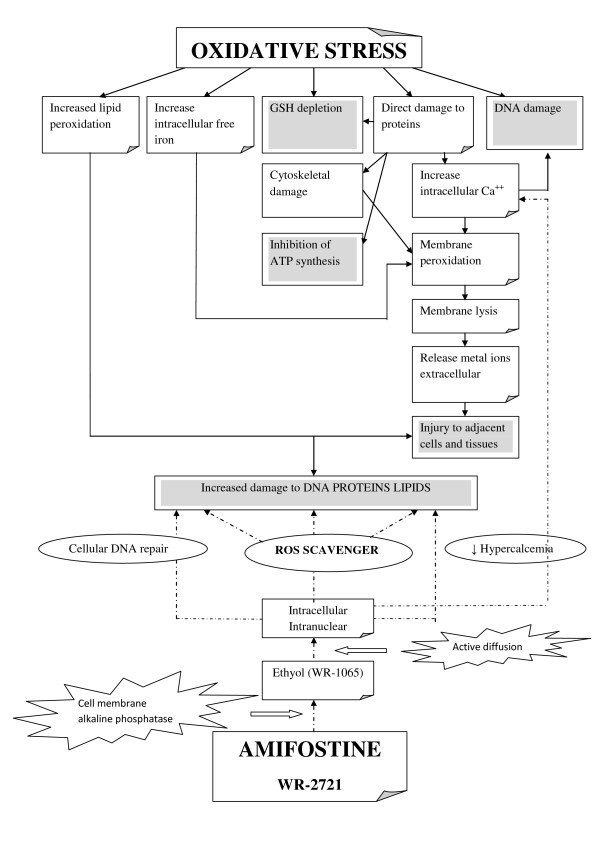
**Oxidative stress and Amifostine**. The diagram demonstrates the cell damage when oxidative stress occurred and the Amifostine protective and repair effects after its activation into cell and nuclear. Straight lines denote the oxidative stress mechanism. Dot lines denote Amifostine points of effect.

The effectiveness of Amifostine appears to be related to its high affinity for DNA, to the similarity in structure of phosphorothioate metabolites to polyamines, and to its effects on processes related to DNA structure and synthesis [33]. Indeed, Amifostine induces the DNA-binding activity of wild-type p53, with its most important biochemical function being the activation of genes involved in control of the cell cycle, apoptosis, cellular differentiation and DNA repair [34,35] (Figure 7). In the present study, we have shown that Amifostine can also be protective in the reduction of oxidative stress induced to the spinal cord cells during ischemia-reperfusion, namely under conditions of descending thoracic or thoraco-abdominal operations. The increase of superoxide radical levels by 27.43% in the spinal cord of ischemic rabbits and a significant 42.68% decrease in the Amifostine group is a direct proof of the development of oxidative stress during aorta occlusion, followed by it's a significant remission after the agent administration. Moreover, oxidative stress was shown indirectly by a 55.3% increase of the lipid peroxidation marker TBARS, followed by a 35.3% significant decrease caused by Amifostine.

Amifostine and its active metabolites seems to be "neuro-protective" factors during spinal cord ischemia, and could be usable in the corresponding operations of thoracic aorta, after clarification (or elucidation) of dose and mode of administration. The time of administration relative to the neuro-cellular damage exposure is critical and the effectiveness of the compound is strongly related to pharmacokinetic properties of the molecule. Having taken into consideration the pharmacokinetic parameters of Amifostine, we managed to achieve the highest concentration of its active metabolite during the time of free radical accumulation in spinal cord cells, by intra-aortic administration, just prior to the release of the aortic occlusion. In our opinion, this maneuver allows the agent to scavenge ROS, as early as possible in their generation, and before the onset of their harmful effect. It is quite interesting to be mentioned that according to the results of the superoxide radical assay, oxidative stress was decreased even below control levels (by 15.25%), suggesting that Amifostine may start its activity against ROS production, before release of aortic occlusion, resulting to maximum spinal cord cell protection.

There are some limitations in our study. This experimental study has been designed as an "acute experiment", focused on the "quantity" of produced oxidative stress of spinal cord, under conditions mimicking descending thoracic aorta operations. We did not design the study for clinical observation of neurologic complications of spinal cord ischemia. It is well known, that these complications can develop several days (till 7) after ischemia, and eventual measurement of oxidative stress at this time, could be unreliable. In addition, the re-agent we used for free radicals detection has an acting-time limitation of 75 minutes. As the positive results of the effectiveness of Amifostine as scavenger of free radicals in spinal cord after ischemia-reperfusion injury have been proved, we have planned the extension of the experiment with focus to the post operative neurological status of the animals.

## Conclusion

In conclusion, the results of our study indicate that intra-aortic Amifostine (WR-2721) infusion during temporary thoracic aorta occlusion has a significant beneficial "neuro-protective" effect in the protection of spinal cord of rabbits. Further studies are needed to clarify the potential application of this "neuro-protective" factor in human beings, during the operations on the descending or thoraco-abdominal aorta.

## Competing interests

The authors declare that they have no competing interests.

## Authors' contributions

All authors: 1) have made substantial contributions to conception and design, or acquisition of data, or analysis and interpretation of data; 2) have been involved in drafting the manuscript or revising it critically for important intellectual content; and 3) have given final approval of the version to be published.
